# Protecting Intestinal Microenvironment Alleviates Acute Graft-Versus-Host Disease

**DOI:** 10.3389/fphys.2020.608279

**Published:** 2021-02-12

**Authors:** Zhengcan Zhou, Ting Shang, Xiurong Li, Hongyan Zhu, Yu-Bo Qi, Xin Zhao, Xi Chen, Zhe-Xin Shi, Guixiang Pan, Yue-Fei Wang, Guanwei Fan, Xiumei Gao, Yan Zhu, Yuxin Feng

**Affiliations:** ^1^State Key Laboratory of Component-based Chinese Medicine, Tianjin University of Traditional Chinese Medicine, Tianjin, China; ^2^Research and Development Center of TCM, Tianjin International Joint Academy of Biotechnology and Medicine, Tianjin, China; ^3^State Key Laboratory of Experimental Hematology, Institute of Hematology & Blood Diseases Hospital, Chinese Academy of Medical Sciences & Peking Union Medical College, Tianjin, China; ^4^First Teaching Hospital of Tianjin University of Traditional Chinese Medicine, Tianjin, China

**Keywords:** acute graft vs. host disease, Xuebijing injection, gut microbiota, allogenic hematopoietic transplantation, cyclosporine A, integrative medicine, biomarkers, Chinese medicine

## Abstract

Acute gut graft-versus-host disease (aGVHD) is a leading threat to the survival of allogeneic hematopoietic stem cell transplantation (allo-HSCT) recipients. Abnormal gut microbiota is correlated with poor prognosis in allo-HSCT recipients. A disrupted intestinal microenvironment exacerbates dysbiosis in GVHD patients. We hypothesized that maintaining the integrity of the intestinal barrier may protect gut microbiota and attenuate aGVHD. This hypothesis was tested in a murine aGVHD model and an *in vitro* intestinal epithelial culture. Millipore cytokine array was utilized to determine the expression of proinflammatory cytokines in the serum. The 16S rRNA sequencing was used to determine the abundance and diversity of gut microbiota. Combining Xuebijing injection (XBJ) with a reduced dose of cyclosporine A (CsA) is superior to CsA alone in improving the survival of aGVHD mice and delayed aGVHD progression. This regimen also reduced interleukin 6 (IL-6) and IL-12 levels in the peripheral blood. 16S rRNA analysis revealed the combination treatment protected gut microbiota in aGVHD mice by reversing the dysbiosis at the phylum, genus, and species level. It inhibited enterococcal expansion, a hallmark of GVHD progression. It inhibited enterococcal expansion, a hallmark of GVHD progression. Furthermore, *Escherichia coli* expansion was inhibited by this regimen. Pathology analysis revealed that the combination treatment improved the integrity of the intestinal tissue of aGVHD mice. It also reduced the intestinal permeability in aGVHD mice. Besides, XBJ ameliorated doxorubicin-induced intestinal epithelial death in CCK-8 assay. Overall, combining XBJ with CsA protected the intestinal microenvironment to prevent aGVHD. Our findings suggested that protecting the intestinal microenvironment could be a novel strategy to manage aGVHD. Combining XBJ with CsA may reduce the side effects of current aGVHD prevention regimens and improve the quality of life of allo-HSCT recipients.

## Highlights

- Combining XBJ with CsA was superior to either CsA or XBJ alone in improving survival and protecting gut microbiota in aGVHD mice.
- Combining XBJ with CsA prevented *E. coli* and enterococcal expansion to ameliorate aGVHD.
- Protecting the intestinal microenvironment is a promising strategy to manage aGVHD.

## Introduction

Preventing acute graft-versus-host disease (aGVHD) saves lives and improves the quality of life of allogeneic hematopoietic stem cell transplantation (allo-HSCT) recipients. As a major target organ of aGVHD, the integrity of the gastrointestinal (GI) tract influences the severity and progression of aGVHD (Hill and Ferrara, 2000; Shono and van den Brink, 2018). Gut microbiota also modulates GVHD and risks of infection in allo-HSCT recipients (Shono and van den Brink, 2018; Stein-Thoeringer et al., 2019).

**GRAPHICAL ABSTRACT:**
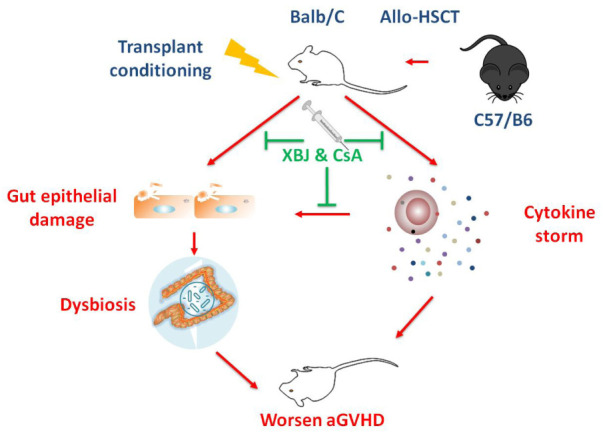
Combination treatment with XBJ and CsA alleviates acute GVHD by protecting the intestinal microenvironment.

Tissue injuries induced by conditioning regimens contribute to the initiation of gut GVHD. In addition, cytokine storms and activated donor-derived cytotoxic T cells worsen gut GVHD (Al-Homsi et al., 2015). Gut microbiota plays an important role in the pathophysiology of GVHD (Shono and van den Brink, 2018). Loss of diversity in gut microbiota is correlated with decreased survival of allo-HSCT recipients (Jenq et al., 2012; Stein-Thoeringer et al., 2019). Increased abundance of *Enterococcus* spp. is correlated with poor clinical outcomes in allo-HSCT recipients (Taur et al., 2012; Holler et al., 2014; Stein-Thoeringer et al., 2019). Fatal *Escherichia coli* infections threaten the survival of patients (DeFilipp et al., 2019). In contrast, butyrate-producing Clostridia, which increased the presence of regulatory T cells (Tregs) in the intestine, are believed to be protective in GVHD development (Shono and van den Brink, 2018; Kumari et al., 2019). Clinical research showed that manipulating gut microbiota [including fecal microbial transplantation (FMT)] may benefit aGVHD patients suffering from gut GVHD (Qi et al., 2018; Fredricks, 2019). Because it is impractical to maintain a decontaminated intestine in allo-HSCT recipients, optimizing gut microbiota becomes a preferred strategy to prevent aGVHD (DeFilipp et al., 2019; Fredricks, 2019).

Intestinal epithelial cells play a cardinal role in maintaining intestinal microenvironment (Artis, 2008). Disrupting intestinal epithelial cells alters the structure of gut microbiota (Kumar et al., 2016; Xiao et al., 2019). The insults of transplant conditioning regimen and aGVHD compromise the integrity of the intestinal microenvironment, which partially contributes to dysbiosis in aGVHD patients (Hanash et al., 2012; Rafei and Jenq, 2020).

Xuebijing injection (XBJ) is a China Food and Drug Administration-approved Chinese medicine injection that contains extracts from five different medicinal herbs [Honghua (*Carthamus tinctorius* flowers), Chishao (*Paeonia lactiflora* roots), Chuanxiong (*Ligusticum chuanxiong* rhizomes), Danggui (*Angelica sinensis* roots), and Danshen (*Salvia miltiorrhiza* roots)] (Jiang et al., 2013; Zhang et al., 2018). It has been used in China to treat multiple organ dysfunction syndromes and sepsis as an add-on to conventional treatments for over a decade (Chen G. et al., 2018; Lyu et al., 2018; Zhang et al., 2018; Zuo et al., 2018; Li et al., 2019). XBJ’s unique feature of cell and organ protection has been revealed by a series of studies (Wang et al., 2007; Li et al., 2014; Xu et al., 2017; Chen X. et al., 2018; Shang et al., 2019). The herbs in XBJ were commonly used to treat different types of human diseases related to organ injuries (Han et al., 2017). Besides, key compounds in XBJ, such as hydroxysafflor yellow A, paeoniflorin, danshensu, salvianolic acid A (SAA), and salvianolic acid B have been shown to protect different organs in various disease models (Liu et al., 2008; Jiang et al., 2014; Hu et al., 2016; Han et al., 2017; Zhu et al., 2018; Shang et al., 2019). However, whether compound Chinese medicine can protect the GI tract to attenuate gut GVHD remains an open question.

In our previous study, we found that a combination of cyclosporine A (CsA) and XBJ is safe and effective in a murine aGVHD model (Lyu et al., 2018). We hypothesized that maintaining the integrity of the intestinal barrier protects gut microbiota and attenuates aGVHD. The influences of an optimized combination regimen of XBJ and CsA on GVHD progression, cytokine production, the barrier function of the intestinal epithelial cells, and the structure of gut microbiota in an aGVHD model were evaluated in this study.

## Materials and Methods

### Chemicals and Reagents

Xuebijing injections (catalog no. z20039833, batch no. 1708221) were purchased from Tianjin Chase Sun Pharmaceutical Co., Ltd. (Tianjin, China). This Chinese medicine is approved by the China Food and Drug Administration (CFDA) for treating sepsis and septic shock (CFDA ratification no. Z20039833). It is routinely used as an add-on to conventional therapy to treat sepsis and septic shock in China (Jiang et al., 2013; Chen X. et al., 2018). This injection contains extracts of five herbs. Each milliliter of Xuebijing is prepared from a combination of 0.1 g each of Honghua (*C. tinctorius* flowers), Chishao (*P. lactiflora* Pall. roots), Chuanxiong (*L. chuanxiong* rhizomes), Danggui (*A. sinensis* roots), and Danshen (*S. miltiorrhiza* roots) (Zhang et al., 2018). Methods of extraction, preparation, and quality control of XBJ were the same as reported previously (Huang et al., 2011; Cheng et al., 2016).

All chemicals used in the experiments were purchased from Sigma-Aldrich (St. Louis, MO, United States) unless specifically indicated. Cytokine detection kit, MILLIPLEX MAP Mouse Th17 Magnetic Bead Panel was ordered from the Merck Millipore Corporation (Billerica, MA, United States). CsA (cat#: SV375) was purchased from Novartis Pharma Stein AG company (Stein, Switzerland).

### Experimental Animals

This study was carried out following the recommendations of the Guide for the Care and Use of Laboratory Animals (NIH publication no. 85–23, revised 1996, United States) and the guidelines of Tianjin University of Traditional Chinese Medicine Animal Research Committee. The protocol was approved by the Tianjin University of Traditional Chinese Medicine Animal Research Committee (TCM-LAE-20170016).

All transplantation experiments were performed with weight-matched (22–24 g) and sex-matched (male) 10-week-old BALB/c, and C57BL/6 mice were purchased from Vital River Company (Beijing, China). Mice were acclimated to the standard germ-free housing room under an ambient temperature of 23°C ± 2°C and 40%–60% relative humidity, with a diurnal cycle of 12-h light and 12-h dark at the animal facility for 1 week before experiments. They were provided with a normal diet and water daily for the duration of experiments.

### aGVHD Model and Bone Marrow Transplantation

A murine aGVHD model was recapitulated following the established protocol, and bone marrow (BM) transplantations were performed as described (Cheng et al., 2013; Al-Homsi et al., 2017b). Briefly, BM cells were gently released from the femurs and tibias of donor C57BL/6 mice and suspended in phosphate-buffered saline (PBS; Fisher Scientific, Waltham, MA, United States). Cell suspensions were then filtered through a 70-μm filter and washed with PBS to obtain particulate-free, single-cell suspensions. GVHD inocula were obtained by gently crushing the spleens of C57BL/6 mice. Splenocytes were then filtered using a 70-μm filter and washed with PBS. Cell counts were performed on hemocytometers. Recipient BALB/c mice were subjected to total body irradiation the day before transplant (day −1). Mice received 8.5 Gy irradiation (two fractions, 3 h apart) via a Rad Source RS-2000 irradiator (San Diego, CA, United States). Irradiated mice received donor BM (5 × 10^6^ cells) with or without splenocytes (1 × 10^7^ cells) by tail-vein injections on day 0. Mice transplanted with BM cells only were used as no GVHD control. Mice receiving BM and splenocytes were randomly divided into following groups: GVHD group (treated with 0.9% NaCl), CsA-treated group [receiving CsA (5 mg/kg, intraperitoneally) alone], XBJ-treated group (0.2 mL/kg, subcutaneously) alone, and combo-treated group (both CsA 2.5 mg/kg, intraperitoneally) and XBJ (0.5 mL/kg, subcutaneously) at the indicated time points. Mice were monitored for weight and scored for GVHD three times weekly. GVHD scoring was based on weight loss, posture, activity, fur texture, skin integrity, and diarrhea and gut injury (severity score 0–2 for each variable, maximum index 12). Animals were euthanized if they lost >35% of their initial weight or reached a score ≥ 7. The experiments were terminated on day 30.

### Ethics Statement

The institutional animal ethics committee approved this study design. Given the severity of our study, we diligently observed all mice to minimize suffering within the frames of the experimental design. All mice in the study were housed in the pathogen-free animal facility, and the overall health status was checked by trained professionals at least two times per day whenever an animal’s condition deteriorated (defined by, among other parameters, decreased activity, progressing hypothermia, rapid weight loss). In detail, mice were euthanized upon signs of impending decease (i.e., inability to maintain upright position/ataxia/tremor and prolonged/deep hypothermia and/or agonal breathing) by cervical dislocation.

### Cytokine Array

Serum samples were collected as described (Al-Homsi et al., 2017a, b). Serum cytokines were measured using the MILLIPLEX multifactor detection technique to simultaneously analyze four inflammatory cytokines. MILLIPLEX MAP Mouse Th17 Magnetic Bead Panel kit (Merck Millipore, Billerica, MA, United States) was used for sample preparations as described (Al-Homsi et al., 2017a, b). The expression of inflammatory factors interleukin 6 (IL-6), IL-12 (P70), IL-23, and tumor necrosis factor α (TNF-α) was detected with a BIO-RAD liquid chip device (Bio-Plex 200 system) (Hercules, CA, United States).

### Fecal Sample Collection and Genomic DNA Extraction

The feces of the experimental mice were obtained in a sterile clean bench. Fresh stool samples from six different mice in each group were collected in sterile tubes and frozen at −80°C. The genomic DNA of the samples was extracted by the CTAB/SDS method [Clark MS (1997) Plant Molecular Biology: A Laboratory Manual. Springer], and then the purity and concentration of the DNA were detected by agarose gel electrophoresis. The genomic DNA was diluted to 1 ng/μL with sterile water and used as a template for polymerase chain reaction (PCR) (Novogene, Beijing, China).

### 16S rRNA PCR and Sequencing

#### Amplicon Generation

16S rRNA genes of distinct regions were amplified using specific primers with the barcode. All PCR reactions were carried out with Phusion High-Fidelity PCR Master Mix (New England Biolabs, Ipswich, MA, United States).

#### PCR Products Mixing and Purification

Polymerase chain reaction products were mixed with the same volume of 1 × loading buffer (contained SYB green) and separated by electrophoresis on 2% agarose gel. PCR products were mixed in equidensity ratios. Then, mixture PCR products were purified with GeneJET Gel Extraction Kit (Thermo Scientific, United States).

#### Library Preparation and Sequencing

Sequencing libraries were generated using Ion Plus Fragment Library Kit 48 rxns (Thermo Scientific) following manufacturer’s recommendations. The library quality was assessed on the Qubit@ 2.0 Fluorometer (Thermo Scientific). The library was sequenced on an Ion S5TM XL platform, and 400-bp/600-bp single-end reads were generated.

### Data Analysis

#### Single-End Reads Quality Control

Single-end reads were assigned to samples based on their unique barcode and were truncated by cutting off the barcode and primer sequence. The raw reads were performed under specific filtering conditions to obtain the high-quality clean reads according to the Cutadapt (V1.9.1^1^) quality-controlled process (Martin, 2011). Afterward, the reads were compared with the reference database (Silva database figurehttps://www.arb-silva.de/) (Quast et al., 2013) using UCHIME algorithm (UCHIME Algorithm^2^) (Edgar et al., 2011) to detect chimera sequences, and then the chimera sequences were removed to obtain the clean reads (Haas et al., 2011).

#### Operational Taxonomic Unit Cluster and Species Annotation

Sequences analysis was performed with Uparse software (Uparse v7.0.1001^3^) (Edgar, 2013). Sequences with ≥97% similarity were assigned to the same operational taxonomic units (OTUs). Representative sequences for each OTU were screened for further species annotation in the Silva Database (version 132) (see text footnote 2) (Quast et al., 2013) based on the Mothur algorithm to annotate taxonomic information. In order to study the phylogenetic relationship of different OTUs, and the difference of the dominant species in different samples (groups), multiple sequence alignment was conducted using the MUSCLE software (version 3.8.31^4^) (Edgar, 2004). OTU abundance information was normalized using a standard of sequence number corresponding to the sample with the least sequences. Subsequent analyses of alpha diversity and beta diversity were all performed basing on this output normalized data.

#### Alpha Diversity

Alpha diversity is applied in analyzing the complexity of species diversity for a sample through six indices, including observed-species, Chao1, Shannon, Simpson, ACE, and Good’s coverage. All these indices in our samples were calculated with QIIME (version 1.7.0) and displayed with R software (version 2.15.3). Two indices were selected to identify community richness: Chao—the Chao1 estimator^5^; ACE—the ACE estimator^6^; two indices were used to identify community diversity: Shannon—the Shannon index^7^; Simpson—the Simpson index^8^; one index to characterized sequencing depth: coverage—the Good’s coverage^9^.

#### Beta Diversity

Beta diversity analysis was used to evaluate differences of samples in species complexity, Beta diversity on both weighted and unweighted UniFrac was calculated by QIIME software (version 1.7.0). Cluster analysis was preceded by principal component analysis, which was applied to reduce the dimension of the original variables using the FactoMineR package and ggplot2 package in R software (version 2.15.3). Principal coordinate analysis (PCoA) was performed to get principal coordinates and visualize from complex, multidimensional data. A distance matrix of weighted or unweighted UniFrac among samples obtained before was transformed to a new set of orthogonal axes, by which the maximum variation factor is demonstrated by the first principal coordinate, and the second maximum one by the second principal coordinate, and so on. PCoA analysis was displayed by the WGCNA package, stat packages and ggplot2 package in R software (version 2.15.3). Unweighted Pair-Group Method with Arithmetic (UPGMA) means clustering was performed as a type of hierarchical clustering method to interpret the distance matrix using average linkage and was conducted by QIIME software (version 1.7.0).

### Hematoxylin–Eosin Staining

The colon tissues obtained from the GVHD and treated groups of mice were fixed in the 10% neutral-buffered formalin and then embedded in paraffin. Subsequently, 5-μm paraffin sections were processed to perform hematoxylin–eosin staining as described before mounting with Pertex (Cui et al., 2017; Chen X. et al., 2018). Colonic mucosa damage scores in different groups of mice were assessed as described (Li et al., 2020).

### Cell Survival Assay

The CCK-8 assay was conducted to evaluate the survival of Caco-2 cells upon doxorubicin and XBJ treatment as described (Chen X. et al., 2018).

### Intestinal Permeability Assay

The assay was conducted as described with modifications (Wu et al., 2019). In brief, intestinal permeability was assessed *in vivo* following oral administration of fluorescein isothiocyanate (FITC)–dextran (7 kDa; Sigma). On day 7, mice were orally gavaged with FITC–dextran (10 mg/20 g). Four hours later, whole blood was obtained by cardiac puncture and centrifuged at 3,500 rpm (1,500 × *g*) for 10 min. Serum was diluted with PBS in 1:1, and fluorescence intensity was measured using excitation at 490 nm and emission at 520 nm with a Tecan microplate reader (Tecan Trading AG, Männedorf, Switzerland). The serum from mice that did not receive FITC–dextran was used as the negative control.

### Statistical Analysis

The log–rank test was used to determine the statistical significance of Kaplan–Meier survival curves. Other results were analyzed by *t* or analysis of variance test as appropriate, using InStat version 3.06 software for Windows (GraphPad, San Diego, CA, United States). The following terminology was used to show statistical significance: ^∗^*P* < 0.05, ^∗∗^*P* < 0.01, and ^∗∗∗^*P* < 0.001.

## Results

### An Optimized Combination Regimen Improved Outcomes in aGVHD Mice

**FIGURE 1:**
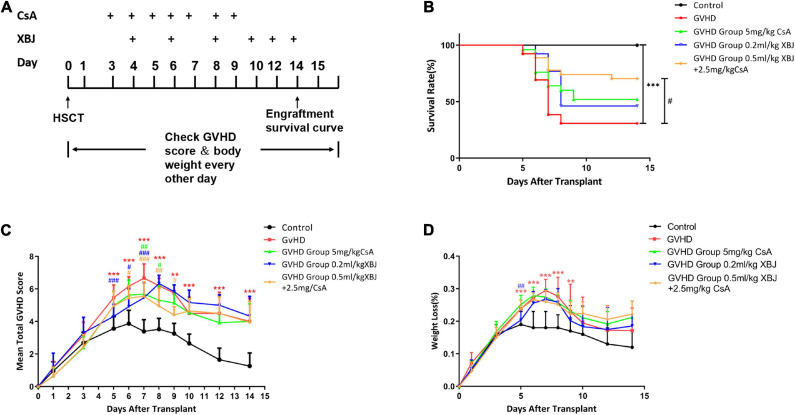
An optimized combination regimen was superior to CsA and XBJ alone in preventing murine aGVHD. **(A)** Experimental scheme. **(B)** Kaplan–Meier survival analysis of the survival in different groups of mice. The log–rank test was performed between different treatment groups and the treatment groups with the GVHD group. The survival rate of each group at day 14 was as follows: control (no GVHD) group: 100%; XBJ and CsA group: 80%; XBJ group: 57.9%; CsA group: 45.4%; GVHD group: 36.8%. **(C)** GVHD scores of different groups of mice. **(D)** Body weight changes after the transplant in different groups of mice. **P < 0.01, ***P < 0.001, GVHD vs. control; ^#^*P* < 0.05, ^##^*P* < 0.01, ^###^*P* < 0.001, treatment groups vs. GVHD (*n* ≥ 13).

In our previous study, 0.2 mL/kg XBJ was identified as the optimal dose of XBJ to prevent/treat GVHD mice individually (Lyu et al., 2018). However, combining 0.2 mL/kg XBJ with 5 mg/kg CsA showed no survival advantage comparing to CsA alone (Lyu et al., 2018). These results triggered us to test different combinations of XBJ and CsA in the murine aGVHD model, aiming to improve the outcome of the combination regimen. The drugs were administered in a new schedule (starting on day 3), and the dosages of XBJ and CsA were also optimized (Figures 1A,B). Combining 0.5 mL/kg XBJ with 2.5 mg/kg CsA (Combo) was superior to other combinations of the two agents (data not shown). We also found that starting the drug administration on day 3 after allo-HSCT yielded better results than administering the regimens on day 1. Interestingly, combining 0.5 mL/kg XBJ with 2.5 mg/kg CsA was superior to 5 mg/kg CsA or 0.2 mL/kg XBJ alone in improving the survival and the GVHD score of GVHD mice (Figures 1B,C). However, the treatments did not significantly impact the bodyweight of GVHD mice (Figure 1D).

### Combining XBJ and CsA Influenced the Expression of Inflammatory Cytokines in aGVHD Mice

**FIGURE 2:**
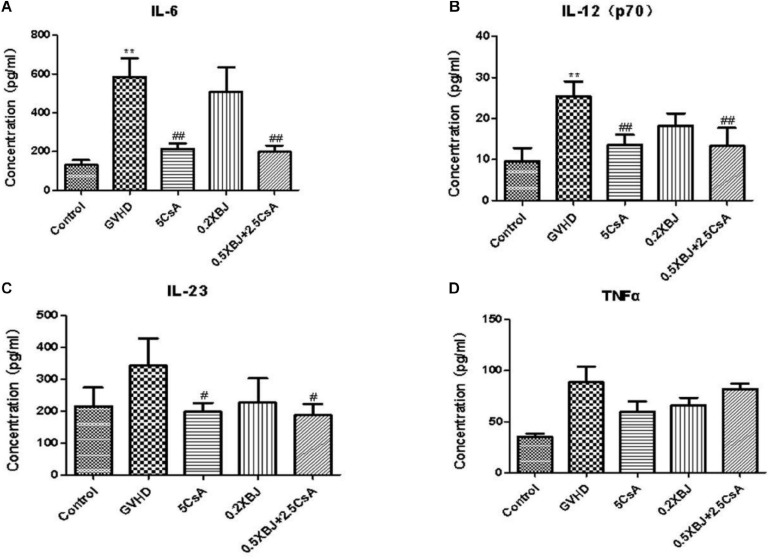
Effects of different treatments on inflammatory cytokines in GVHD mice. On the seventh day after transplantation, serum from different groups of mice was collected and subjected to cytokine array analysis. Statistical analysis of IL-6 **(A)**, IL-12(p70) **(B)**, IL-23 **(C)**, and TNF-α **(D)** was presented. *The significant difference between control and GVHD groups; ^#^the significant difference between each treatment group and the GVHD group. **P < 0.01, ^#^*P* < 0.05, ^##^*P* < 0.01. *n* = 4–6/group.

We also determined the effects of different treatments on the expression of inflammatory cytokines in aGVHD mice. CsA significantly inhibited the expression of IL-6, IL-12p70, and IL-23 (Figures 2A–C). The combination regimen showed similar effects on cytokine expression as the CsA treatment group (Figures 2A–C). The expression of TNF was not significantly impacted by XBJ or CsA (Figure 2D).

### XBJ and CsA Attenuated Dysbiosis in aGVHD Mice

**FIGURE 3:**
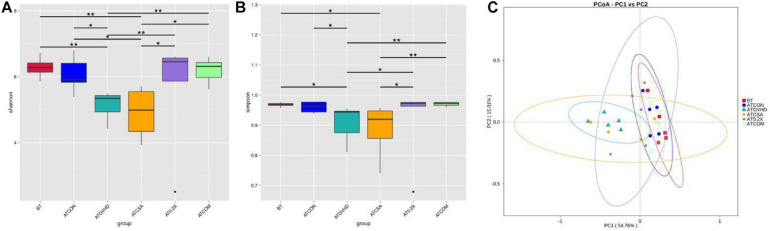
Combination treatment normalized gut microbiota of aGVHD mice. **(A)** Shannon index of alpha diversity was examined by 16S high-throughput sequencing on day 7 in the GVHD, control, and different treatment groups. *n* = 4–6/group. Statistical significances are indicated: The Wilcoxon rank-sum test. The top and bottom boundaries of each box indicate the 75th and 25th quartile values, respectively, and lines within each box represent the 50th quartile (median) values. Ends of whiskers mark the lowest and highest diversity values in each instance. **(B)** Simpson index of alpha diversity was examined by 16S high-throughput sequencing on day 8 in GVHD, control, and different treatment groups. *n* = 4–6/group. **(C)** Weighted UniFrac-based principal coordinates analysis (PCoA). The principal component was used to measure the shift of the intestinal bacterial composition structure in different groups of mice after BM transplantation at day 8. *n* = 4–6/group. BT, samples from mice without BM transplantation; ATCON, samples from BALB/c mice transplanted with BM from C57 donors only; ATGVHD, samples from BALB/c mice transplanted with BM and splenocytes from C57 donors; ATCSA, samples from BALB/c mice transplanted with BM and splenocytes from C57 donors treated with 5 mg/kg CsA; AT0.2X, samples from BALB/c mice transplanted with BM and splenocytes from C57 donors treated with 0.2 mL/kg XBJ; ATCOM, samples from BALB/c mice transplanted with BM and splenocytes from C57 donors treated with 2.5 mg/kg CsA and 0.5 mL/kg XBJ. ^*,**^Compared with aGVHD group.

To determine how the combination treatment may impact the progression of aGVHD, 16S rRNA sequencing was conducted to evaluate the abundance of gut microbiota in different groups of mice on day 7 after the transplantation. aGVHD caused persistent dysbiosis at day 7, and the combination treatment effectively attenuated these changes. Shannon and Simpson’s diversity index revealed significant differences between the combo group (2.5 mg/kg CsA-treated and 0.5 ml/kg XBJ-treated groups) and the GVHD group (Figures 3A,B). PCoA was used to further determine the influence of the different treatments on the gut microbiota profiles in aGVHD mice. The gut bacterial composition profile of aGVHD mice changed substantially on day 7 after allo-HSCT, and this change was inhibited by the combo treatment (*p* = 0.006). In contrast, the combo-treated group showed similar gut microbiota profiles as the no-GVHD group (ATCON group) and untransplanted groups (BT group) (Figure 3C). There was no significant difference between the no-GVHD group (ATCON) and the combo-treated group in analysis of similarities (*p* = 0.078).

### Combination Treatment Reversed Abnormal Gut Microbiota in aGVHD Mice at the Phylum Level

**FIGURE 4:**
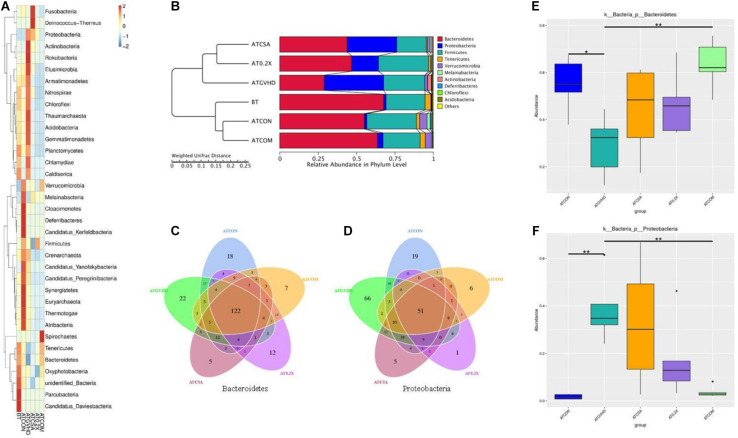
The combination of XBJ and CsA reversed the dysbiosis at the phylum level in GVHD mice. **(A)** The cluster heat map of the abundance of gut microbiota at the phylum level in different groups of mice. The alteration of intestinal bacterial patterns at the phylum level in different groups of mice was assessed using 16S high-throughput sequencing after BMT on day 7, *n* = 4–6/group. The heat map is color-based on row *Z* scores. The mice with the highest and lowest bacterial level are in red and blue, respectively. **(B)** Unweighted Pair-Group Method with Arithmetic (UPGMA) mean tree based on weighted UniFrac at the phylum level. The relative abundances of enteric bacteria at the phylum level in different groups of mice were assessed using 16S high-throughput sequencing after BMT on day 7. *n* = 4–6/group. The proportion of relative abundance of Bacteroidetes and Proteobacteria was decreased in aGVHD mice compared to control mice, and the combination treatment reversed this change. **(C)** OTU-based Venn graph in Bacteroidetes of different groups. The number of OTUs in the phylum of Bacteroidetes in different groups was analyzed using 16S high-throughput sequencing after BMT at day 7. *n* = 4–6/group. **(D)** OTU-based Venn graph in Proteobacteria of different groups. The number of OTUs in the phylum of Proteobacteria in different groups was analyzed using 16S high-throughput sequencing after BMT on day 7. *n* = 4–6/group. **(E)** MetaStat analysis of Bacteroidetes in different groups to evaluate the influences of treatments on the relative abundance. All values are mean ± SEM (*n* = 4–6/group). *Adjusted *p* < 0.05, **adjusted *p* < 0.01. **(F)** MetaStat analysis of Proteobacteria of different groups to evaluate the influences of treatments on the relative abundance of Bacteroidetes. All values are mean ± SEM (*n* = 4–6/group). *Adjusted *p* < 0.05, **adjusted *p* < 0.01.

We analyzed the impact of the treatments on the gut microbiota of aGVHD mice at the phylum level. Irradiation exposure and aGVHD drastically disturbed the relative abundance of intestinal flora at the phylum level (Figure 4A). The combo treatment partially reversed the abnormality in aGVHD mice, comparing with the no-GVHD groups (Figure 4A). Irradiation exposure and aGVHD caused a down-regulation of the relative abundance of Bacteroidetes (or Firmicutes) and an increase of Proteobacteria at the phylum level in aGVHD mice at day 7. The combo treatment reversed these changes (Figures 4A,B). Venn analysis revealed that combo treatment changed the composition of OTUs in the phylum of Bacteroidetes and Proteobacteria in aGVHD mice (Figures 4C,D). Importantly, the relative abundances of the two phyla were also normalized by the combo treatment in MetaStat analysis comparing to GVHD mice (Figures 4E,F). Together, these results demonstrated that the combo treatment preserves the gut bacterial composition in aGVHD mice.

### Combining XBJ With CsA Normalized Gut Microbiota of aGVHD Mice at the Genus Level

**FIGURE 5:**
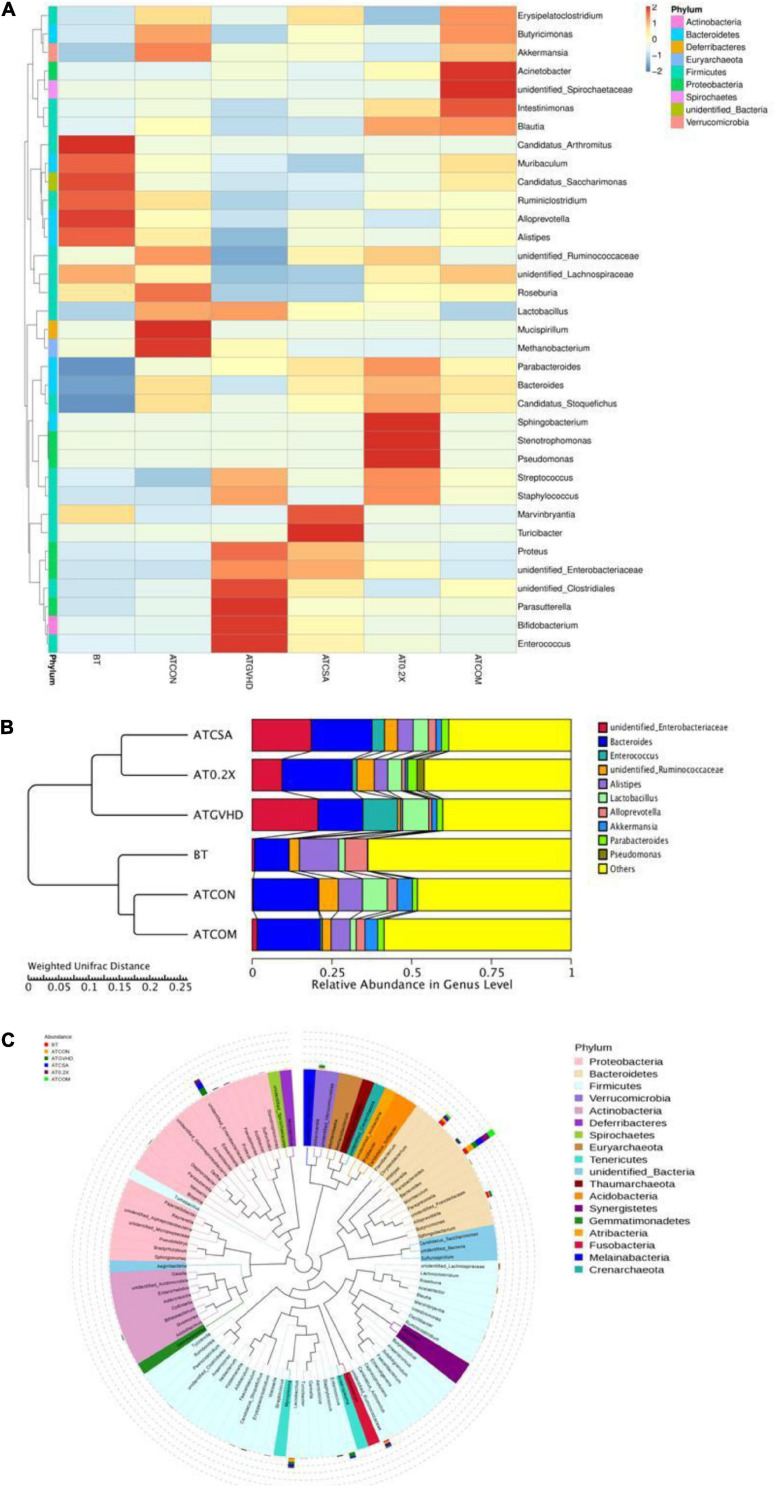
Combination therapy rescued the disordered gut microbiota in GVHD mice at the genus level. **(A)** Cluster heat map of relative abundance of gut microbiota at the genus level in different groups of mice. The alteration of intestinal bacterial patterns at the genus level in different groups of mice was assessed using 16S high-throughput sequencing after BMT at day 7, *n* = 4–6/group. The heat map is color-based on row *Z* scores. The mice with the highest and lowest bacterial level are in red and blue, respectively. **(B)** The UPGMA tree based on weighted UniFrac distances in the genus level. The relative abundances of enteric bacteria at the genus level in different groups of mice were assessed using 16S high-throughput sequencing after BMT on day 7. *n* = 4–6/group. **(C)** Classification of the top 100 genera in the abundance of the gut microbiota and the dominant genera in different groups of mice. Dominant genera in different groups of mice were marked with indicated colors. Bar lengths indicated the abundance of the genus in different groups of mice.

Next, we analyzed the 16S rRNA sequencing results at the genus level to determine the influence of different treatments on the gut microbiota of aGVHD mice. We found the combo treatment altered the relative abundances of the top 35 genera, comparing with GVHD, CsA-treated, and XBJ-treated groups. The overall abundance of the top 35 genera in the combo group was similar to the no-GVHD groups comparing to CsA- and XBJ-treated group. *Enterococcus*, a biomarker of GVHD, was reversed by the combo treatment to a similar level as those in no-GVHD (ATCON) and non-transplanted (BT) mice (Figure 5A). Other genera, such as Akkermansia, Bacteroides, Parasutterella, and unidentified Clostridiales, were also normalized to the level of no-GVHD and non-transplanted mice.

The UPGMA tree analysis revealed the relative abundances of unidentified Enterobacteriaceae, unidentified Ruminococcaceae, and Alistipes were significantly decreased in aGVHD mice compared to control mice. Enterococcal expansion is a hallmark of dysbiosis in aGVHD (Stein-Thoeringer et al., 2019). Of note, significant enterococcal expansion in aGVHD mice was also unveiled in this study (Figures 5A,B). The combination treatment reversed all these changes (Figure 5B and Table 1). Four genera were identified as potential biomarkers in aGVHD mice that were influenced by the combo treatment, including Alistipes, unidentified Enterobacteriaceae, unidentified Ruminococcaceae, and *Enterococcus* (Figure 5C, Supplementary Figure 3, and Table 1).

**TABLE 1:** The influences of different treatments on the gut microbiota of aGVHD mice on genus/species level.

Effect	Genus/Species	CSA	0.2X	COM
Up-regulated	Alistipes	–	*P* < 0.01	*P* < 0.01
	(UR)	–	*P* < 0.05	*P* < 0.05
Down-regulated	(UE)	–	–	*P* < 0.01
	*Enterococcus*	–	*P* < 0.05	*P* < 0.05
	*Escherichia coli*	–	–	*P* < 0.01
	*Enterococcus durans*	–	*P* < 0.05	*P* < 0.05

### Treatment With XBJ and CsA Normalized the Abundance of *Enterococcus durans* and *E. coli* in aGVHD Mice

**FIGURE 6:**
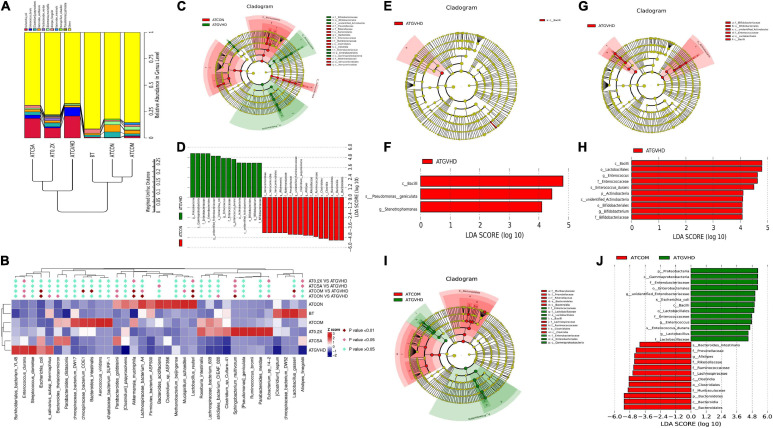
Combination treatment reversed the dysbiosis in GVHD mice on the species level. **(A)** The UPGMA tree of the top 10 species in abundance based on weighted UniFrac distances in the species level. The relative abundances of enteric bacteria at the species level in different groups of mice were assessed using 16S high-throughput sequencing after BMT on day 7. *n* = 4–6/group. **(B)** The statistical analysis of the abundance of top 35 species in gut microbiota between different groups of mice. *n* = 4–6/group. The heat map is color-based on row *Z* scores. The groups with the highest and lowest bacterial level are marked in red and blue, respectively. The combination treatment significantly down-regulated the relative abundance of *E. coli* and *Enterococcus durans*. **(C–J)** Cladograms **(C,E,G,I)** and LDA (linear discriminant analysis) score **(D,F,H,J)** generated by LEfSe (linear discriminant analysis effect size) indicating differences in bacterial taxa in different groups of mice 7 days after the BMT. *n* = 4–6/group. The central point represents the root of the tree (bacteria), and each ring represents the next lower taxonomic level (from phylum to genus). The diameter of each circle represents the relative abundance of the taxon. Only the taxa meeting a significant LDA threshold value of >2 are shown. The species in the non-transplant group (ATCON) and the combo treatment group (ATCOM) were indicated with a negative LDA score **(D,J)**. **(C,D)** LEfSe analysis of the ATCON vs. the GVHD group. The potential biomarkers of the GVHD group: (1). Proteobacteria, Gammaproteobacteria, Enterobacteriales, Enterobacteriaceae, unidentified Enterobacteriaceae, *E. coli*; (2). Firmicutes, Bacilli, Lactobacillales, Enterococcaceae, *Enterococcus*, *E. durans*. The potential biomarkers of the ATCON group: (1). Bacteroidetes, Bacteroidia, Bacteroidales, Rikenellaceae, *Alistipes*; (2). Firmicutes, Clostridia, Clostridiales, Ruminococcaceae, unidentified Ruminococcaceae, *Clostridium papyrosolvens*; (3). Verrucomicrobia, Verrucomicrobiae, Verrucomicrobiales, Akkermansiaceae, Akkermansia. **(E,F)** The LEfSe analysis of the GVHD vs. the CSA group. **(G,H)** The LEfSe analysis of the GVHD vs. the 0.2 mL/kg XBJ-treated group. **(I,J)** The LEfSe analysis of the GVHD vs. COM group.

We further analyzed how the combo treatment impacts gut microbiota on species level in GVHD mice. *E. coli* and *Enterococcus durans* were normally in low abundance in non-transplanted and no-GVHD mice. However, they became the top two species in relative abundance (among the top 10 high abundance species) in GVHD mice (Figure 6A). The combo treatment reversed the expansion of both species in GVHD mice (Figures 6A,B).

The relative abundance of *Clostridium papyrosolvens* was significantly decreased in aGVHD mice. The Combo treatment reversed this abnormality (Figure 6B). Notably, the combo, BT, and ATCON groups were separated from the aGVHD group and the CsA group in the hierarchical cluster tree analysis (Figure 6A). These results were also confirmed by comparing the relative abundances of the top 35 species among different groups (Figure 6B). The combo treatment dramatically reduced the abundance of *E. durans* and *E. coli*. LDA effect size (LEfSe) analysis revealed that the combo group vs. GVHD had similar biomarkers as the no-GVHD group vs. GVHD group (Figures 6D,E,J). In contrast, the CsA-treated group vs. GVHD and XBJ vs. GVHD group showed different sets of biomarkers on the species level (Figures 6F–I).

### XBJ Protected Intestinal Tissue in aGVHD Mice

**FIGURE 7:**
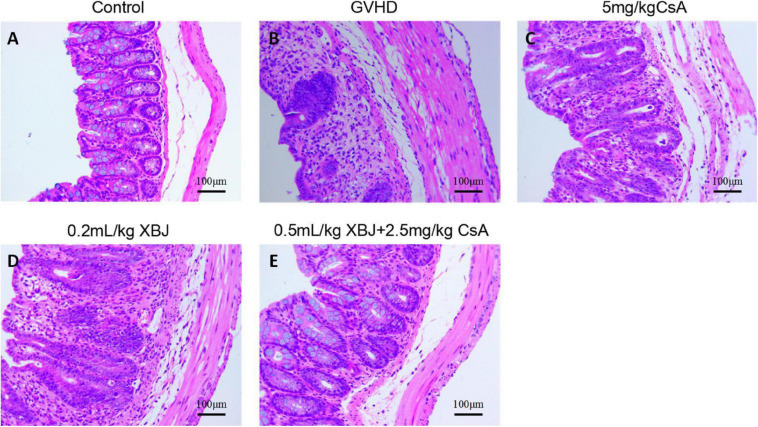
XBJ protected intestinal tissue in aGVHD mice. Representative hematoxylin–eosin staining of colon tissues in different groups of mice was presented. **(A)** No-GVHD control group; **(B)** GVHD group; **(C)** 0.2 mL/kg XBJ-treated group; **(D)** 5 mg/kg CsA-treated group; **(E)** 0.5 mL/kg XBJ and 2.5 mg/kg CsA-treated group. Scale = 100 μm. *n* = 4–6/group.

**FIGURE 8:**
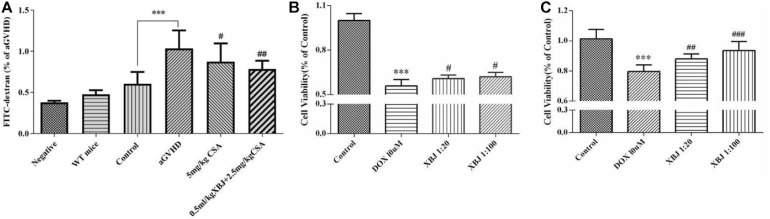
XBJ maintained the integrity of the gut microenvironment and prevented doxorubicin (DOX)-induced cell death in Caco-2 cells. **(A)** Gut permeability assay to determine the influence of the combination therapy on the integrity of the intestine tissue of aGVHD mice. FITC–dextran concentrations (% of aGVHD) in serum were measured on day 7 after the transplant. Data are means ± SD. Data were analyzed using ordinary one-way analysis of variance for multiple comparisons. *Control compared with aGVHD, ***P < 0.001; ^#^treatment groups compared with aGVHD; ^#^*P* < 0.05, ^##^*P* < 0.01, ^###^*P* < 0.001. **(B,C)** The CCK-8 assay was used to determine the survival of Caco-2 cells in the presence of doxorubicin and XBJ. **(B)** Caco-2 cells were pretreated with XBJ as indicated for 24 h and were then treated with DOX for 12 h before subjecting to CCK-8 assay. **(C)** Caco-2 cells were treated with DOX for 12 h, and XBJ was added to the indicated groups for 24 h before subjecting to CCK-8 assay. Each experiment was repeated at least three times. *Control vs. DOX-treated groups; ***P < 0.001; ^#^XBJ-treated groups vs. DOX-treated group; ^#^*P* < 0.05, ^##^*P* < 0.01, ^###^*P* < 0.001.

We further analyzed the histology of the intestine in different groups of mice. The combo-treated group showed relative normal villi morphology, comparing with the GVHD group, which showed reduced numbers of villi (Figures 7A–E), indicating XBJ may protect the intestines of aGVHD mice. Consistently, intestinal permeability assay revealed that combo treatment reduced intestinal permeability of aGVHD mice, indicating XBJ may improve the integrity of intestinal tissue (Figure 8A). To determine whether XBJ exerted tissue protection in the intestine, Caco-2 cells were treated with doxorubicin at the presence and absence of XBJ. XBJ improved the survival of Caco-2 cells in the CCK-8 assay, suggesting XBJ can attenuate intestinal injuries in aGVHD mice (Figures 8B,C).

## Discussion

### Highlights of This Study

We found that combining XBJ with the reduced dose of CsA is superior to CsA alone in preventing aGVHD. It reduced proinflammatory cytokine production and protected the gut microbiota of aGVHD mice. Specifically, combo treatment reversed *E. coli* and *E. durans* expansion in aGVHD mice. XBJ may protect the intestinal tissue of GVHD mice to prevent dysbiosis.

### Combination Therapy Improved Outcomes in a Murine aGVHD Model

In our previous work, combining 0.2 mL/kg XBJ with 5 mg/kg CsA was safe and effective in rescuing mice from lethal aGVHD. However, there was no significant difference between the combined regimen and the CsA alone group in the survival (Lyu et al., 2018). Five milligrams per kilogram CsA was used as a standard dose for experimental GVHD prevention (Li et al., 2013; Yuan et al., 2015). Combining different doses of XBJ (from 0.2 to 0.5 mL/kg or higher) with 5 mL/kg CsA did not translate into better outcomes (data not shown), suggesting 5 mg/kg CsA may maximize its effect on preventing aGVHD in the mouse model. We reasoned that adjusting the doses of XBJ and CsA while shifting the schedule of drug administration may yield a better outcome. After testing different combinations of XBJ and CsA at various schedules (data not shown), we found combining 0.5 mL/kg XBJ with 2.5 mg/kg CsA is superior to 5 mg/kg CsA alone in improving the survival and reversing dysbiosis of gut microbiota in aGVHD mice. Side effects of CsA compromise the quality of life in allo-HSCT recipients (Gijtenbeek et al., 1999; Vitko and Viklicky, 2004). Our results indicated this combo regimen may reduce the side effects of CsA (such as causing kidney failure) in the long term.

### Potential Mechanisms: Combo Treatment Limited Proinflammatory Cytokine Production

Proinflammatory cytokines promote the progression of aGVHD (Bastian et al., 2019; Zeiser, 2019). IL-6, IL-12, and IL-23 contribute to the worse outcomes in aGVHD. Clinical trials exploring targeting IL-12/IL-23 and IL-6 inhibition in prophylaxis and treatment of GVHD are ongoing (Tvedt et al., 2017; Hill and Koyama, 2020). The combo treatment may prevent aGVHD by inhibiting the production of proinflammatory cytokines in aGVHD mice. Similar to 5 mg/kg CsA treatment, combining 0.5 mL/kg XBJ with 2.5 mg/kg CsA reduced serum proinflammatory cytokines (including IL-6, IL-23, and IL-12) in aGVHD mice, indicating XBJ may play a role in reducing the production of inflammatory cytokines in aGVHD mice (Tvedt et al., 2017; Bastian et al., 2019).

### Potential Mechanisms: Combo Treatment Reversed Dysbiosis on Phylum, Genus, and Species Levels

Increasing evidence suggests gut microbiota plays an important role in the progression of aGVHD (Shono and van den Brink, 2018; Fredricks, 2019; Stein-Thoeringer et al., 2019). Conditioning regimens and activated donor-derived T cells may damage the GI tract (Al-Homsi et al., 2015; Staffas et al., 2017). This may lead to dysbiosis in allo-HSCT recipients, which worsens GVHD. Interventions that restore normal gut microbiota may serve as a therapeutic option for GI tract aGVHD (Kakihana et al., 2016; Qi et al., 2018). Preventing the dysbiosis of gut microbiota in allo-HSCT recipients reduces the risk of severe GVHD. Our 16S rRNA sequencing results revealed that combining XBJ with CSA is superior to 5 mg/kg CSA or XBJ alone in maintaining gut microbiota on phyla, genera, and species levels. Dominating gut microbiota by *E. coli* and *Enterococcus* were frequently observed in allo-HSCT recipients (Jenq et al., 2012; Shono and van den Brink, 2018). The expansion of *Enterococcus* promotes the progression of aGVHD in mice (Stein-Thoeringer et al., 2019). Consistent with the clinical observation, we found that *E. coli* and *E. durans* became the dominant species in aGVHD mice (Figure 6). However, the combo treatment reversed this phenomenon, indicating XBJ may maintain the integrity of the GI tract to protect gut microbiota.

Clinical and experimental studies showed allo-HSCT results in the reduced abundance and diversity of gut microbiota in patients and animal models (Jenq et al., 2012; Taur et al., 2012; Shono and van den Brink, 2018; Stein-Thoeringer et al., 2019). Increased abundance of *Enterococcus* spp. is a poor prognosis marker of aGVHD. Stein-Thoeringer et al. reported that the enterococcal expansion in human allo-HSCT recipients was associated with a significant reduction of survival and increased GVHD-related mortality in a multicenter and a single-center clinical study (Stein-Thoeringer et al., 2019). It increased the possibility of bloodstream infection, which threatens the survival of allo-HSCT recipients (Taur et al., 2012), compromising epithelial barrier integrity (Steck et al., 2011) and stimulating TNF production from macrophages (Kim et al., 2006). Although *E. durans* was considered as a low-virulence organism, it may cause a fatal outcome in patients with advanced diseases despite optimized antibiotic therapy (Vijayakrishnan and Rapose, 2012). Reversing the expansion of *Enterococcus* spp. with the combo treatment partially explained the mechanism of XBJ on preventing aGVHD.

The role of *E. coli* in aGVHD progression remains to be determined. *E. coli* may produce indole to prevent GVHD (Swimm et al., 2018). Eriguchi et al. (2012) found an association between the expansion of *E. coli* and worse GVHD in a preclinical study. Some *E. coli* strains are pathogenic and can cause lethal infection (Fukuda et al., 2011; DeFilipp et al., 2019). Overall, the combo treatment is more effective than CSA alone in protecting gut microbiota.

### Mechanisms of the Combo Treatment on Gut Microbiota Protection

Improving the integrity of the intestinal tissue is associated with better outcomes in aGVHD (Joly et al., 2016). GVHD induces donor T cell-dependent and independent epithelial death in preclinical models. However, there is no effective intervention to manage allo-HSCT and GVHD-related epithelial death/intestinal injuries (Hanash et al., 2012).

We did not detect a significant difference in the gut microbiota between the 5 mg/kg CsA-treated group and GVHD group on phylum, genus, and species level in our study (Figures 4B,F, 5B, 6A,B). In contrast, combining XBJ and CsA showed dramatic impacts on the gut microbiota of GVHD mice. Consistent with our results, O’Reilly et al. (2020) concluded that CsA at the clinically relevant doses had negligible direct effects on the gut microbiota composition *ex vivo* and in healthy volunteers. However, Jia et al. (2019) reported that CSA ameliorates hepatic graft injury and partially restores gut microbiota in a rat orthotopic liver transplantation model. These differences may relate to drug delivery and disease models.

Xuebijing injection may directly or indirectly influence the abundance of gut microbiota in aGVHD mice. Our histological analysis of colon tissues in different groups of mice indicated that the combo treatment maintained the morphology of intestine epithelial cells better than individual agents, suggesting XBJ may protect the intestine in aGVHD mice to normalize gut microbiota indirectly (Figure 7). Additionally, the combo treatment reduced the permeability of the intestine in aGVHD mice (Figure 8A). XBJ rendered protection to gut epithelial cells in the presence of doxorubicin *in vitro* (Figures 8B,C). In our previous study, XBJ treatment enhanced Treg differentiation *in vitro* (Chen X. et al., 2018). The combo treatment may influence the Treg population in the intestine to protect the intestinal epithelial cells.

Whether XBJ can directly influence the abundance of gut microbiota is still an open question. Our *in vitro* culture experiment revealed that XBJ does not affect bacteria growth (data not shown), suggesting that the indirect influence of XBJ on gut microbiota may play a major role in aGVHD mice.

### Chinese Medicine in Managing Acute Gut GVHD

Limited studies were conducted to study the influence of Chinese medicine injections on gut microbiota. Recent research found that tail-vein injection of *S. miltiorrhiza* (Danshen) extract and salvianolic acid A, a compound in Danshen and XBJ, protected the intestine in rodent models of gut injuries (Wen et al., 2013; Wang et al., 2018). Other herbs in XBJ, such as *Honghua*, *Chuanxiong*, and *Chishao*, showed tissue protection effects in animal models and clinical studies. Besides, key compounds in these medical herbs, such as paeoniflorin and gallic acids, showed similar cell- and tissue-protective effects as the extracts from the herbs (Zhang et al., 2014; Gu et al., 2017; Zhu et al., 2019). These results suggested that XBJ maintains the intestinal microenvironment by protecting the intestinal epithelial barrier. In our previous study, C0127 (containing four active compounds in XBJ) simulated the effects of XBJ in preventing systemic *Candida albicans* infection and *C. albicans*-induced kidney failure (Shang et al., 2019). These four compounds, including hydroxysafflor yellow A and paeoniflorin, may play a key role in preventing aGVHD when combining with CsA.

### The Potential Advantages of the Combo Treatment in the Clinic

In the clinic, antibiotics significantly reduced the diversity and abundance of gut microbiota in allo-HSCT recipients. Reducing the use of antibiotics while preventing bacteremia/sepsis in allo-HSCT recipients remains a challenge (Shono and van den Brink, 2018). However, our results indicate that the application of XBJ in aGVHD prophylaxis may kill two birds with one stone. As a safe alternative to antibiotics, XBJ may prevent bacteremia/sepsis while protecting gut microbiota in allo-HSCT recipients (Liu et al., 2015; Chen X. et al., 2018). Side effects of CsA and resistance to CsA limited its benefits to allo-HSCT recipients (Gijtenbeek et al., 1999; Vitko and Viklicky, 2004). Combining XBJ with low-dose CsA may improve the quality of life by reducing side effects of CsA and the risk of CsA resistance.

## Conclusion

In summary, combining XBJ with CsA is superior to CsA alone in preventing lethal aGVHD. Our limited evidence indicated that XBJ may protect the epithelial barrier to attenuate aGVHD. The combo regimen protected gut microbiota and reversed the abnormal expansion of *E. coli* and *E. durans* in the intestine of aGVHD mice. XBJ may protect intestinal tissue to prevent dysbiosis. This pilot study provided proof-of-concept evidence that protecting the intestinal microenvironment may shed light on the management of acute gut GVHD. However, signaling pathways regulated by XBJ in gut epithelial cells remain to be studied. Whether XBJ regulates inflammation to protect organs is also an open question. The material base of XBJ in protecting gut microbiota remains to be unrevealed.

## Data Availability Statement

All datasets generated for this study are included in the article/Supplementary Material.

## Ethics Statement

The animal study was reviewed and approved by Tianjin University of Traditional Chinese Medicine Animal Research Committee.

## Author Contributions

YF and YZ conceptualized the ideas of this work. YF, ZZ, TS, XL, Y-BQ, and XZ carried out the experiments. YF, ZZ, and HZ wrote the original draft paper. YF, HZ, XC, XG, Z-XS, XZ, GP, TS, GF, and YZ analyzed the experimental results and revised the paper. YF, YZ, GF, Y-FW, and XG supervised the work and contributed to the study conceptualization. All authors contributed to the article and approved the submitted version.

## Conflict of Interest

The authors declare that the research was conducted in the absence of any commercial or financial relationships that could be construed as a potential conflict of interest.

## Footnotes

1 http://cutadapt.readthedocs.io/en/stable/

2 http://www.drive5.com/usearch/manual/uchime_algo.html

3 http://drive5.com/uparse/

4 http://www.drive5.com/muscle/

5 http://www.mothur.org/wiki/Chao

6 http://www.mothur.org/wiki/Ace

7 http://www.mothur.org/wiki/Shannon

8 http://www.mothur.org/wiki/Simpson

9 http://www.mothur.org/wiki/Coverage

## Supplementary Material

Supplementary Figure 1 Sequencing depth and species diversity of the 16S rRNA sequencing. **(A)** Species accumulation boxplot of samples. With the increase of sample numbers, observed species gradually increase and finally flat. **(B,C)** Rarefaction curve of samples. Sequencing depth reached 60,000–80,000 sequences. **(D,E)** The rank abundance of samples. The abundance between species is not much different, and species distribution is uniform in the samples. *n* = 4–6/group.

Supplementary Figure 2 Alpha diversity indexes of different groups the 16S rRNA sequencing. **(A)** Observed species index. OTU numbers in aGVHD mice were significantly decreased. **(B)** PD whole tree index. Species’ genetic relationship in aGVHD mice becomes simple. **(C)** ACE index. **(D)** Chao1 index. Panels **(C,D)** revealed that species numbers were significantly reduced in aGVHD mice. *n* = 4–6/group, **p* < 0.05, ***p* < 0.01.

Supplementary Figure 3 The statistic analysis of the abundance of top 35 genera between different groups of mice. The heat map is color-based on row *Z* scores. The highest and lowest bacterial abundance in each genus was marked with red and blue, respectively. The combination treatment significantly up-regulated the relative abundance of Alistipes, unidentified Ruminococcaceae, and Akkermansia. The relative abundance of Enterobacteriaceae and *Enterococcus* were down-regulated in mice receiving the combo treatment. *n* = 4–6/group.

Supplementary Figure 4 Histopathological scores of colons in different groups of mice. *n* = 4–6/group, **p* < 0.05, Control vs aGVHD group; #*p* < 0.05, compared with aGVHD group.

## Abbreviations

XBJ: Xuebijing injection
CsA: cyclosporine A
aGVHD: acute graft-versus-host disease
allo-HSCT: allogeneic hematopoietic stem cell transplantation
FMT: fecal microbial transplantation
GI: gastrointestinal
