# Supplementary figures and images for: Protecting Intestinal Microenvironment Alleviates Acute Graft-Versus-Host Disease
Source: Front Physiol. 2021 Feb 12;11:608279. doi: 10.3389/fphys.2020.608279 (PMC7907526; doi:10.3389/fphys.2020.608279)

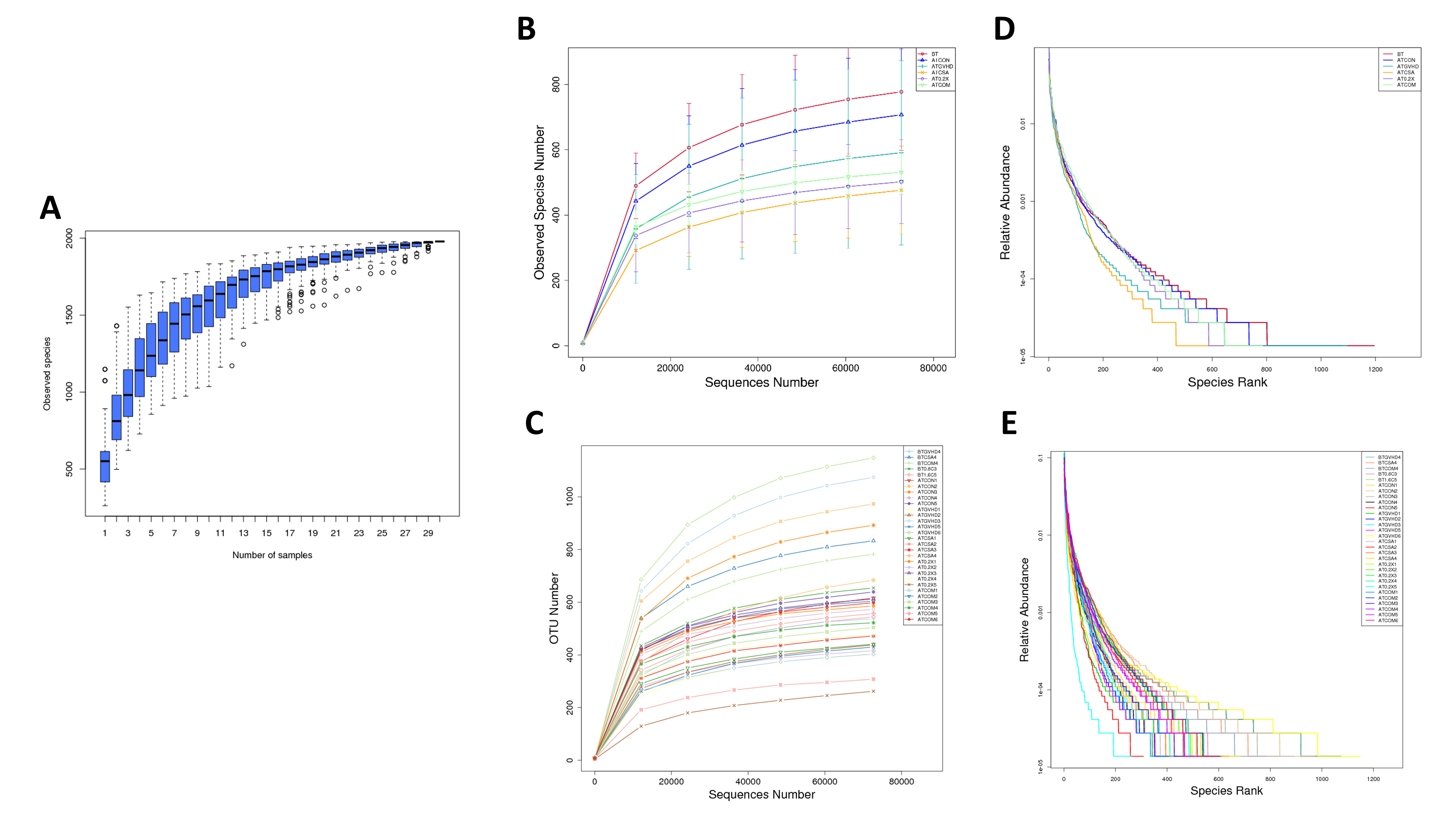

Supplement: Supplementary Figure 1 — Sequencing depth and species diversity of the 16S rRNA sequencing. (A) Species accumulation boxplot of samples. With the increase of sample numbers, observed species gradually increase and finally flat. (B,C) Rarefaction curve of samples. Sequencing depth reached 60,000–80,000 sequences. (D,E) The rank abundance of samples. The abundance between species is not much different, and species distribution is uniform in the samples. n = 4–6/group. [file Image_1.tif]

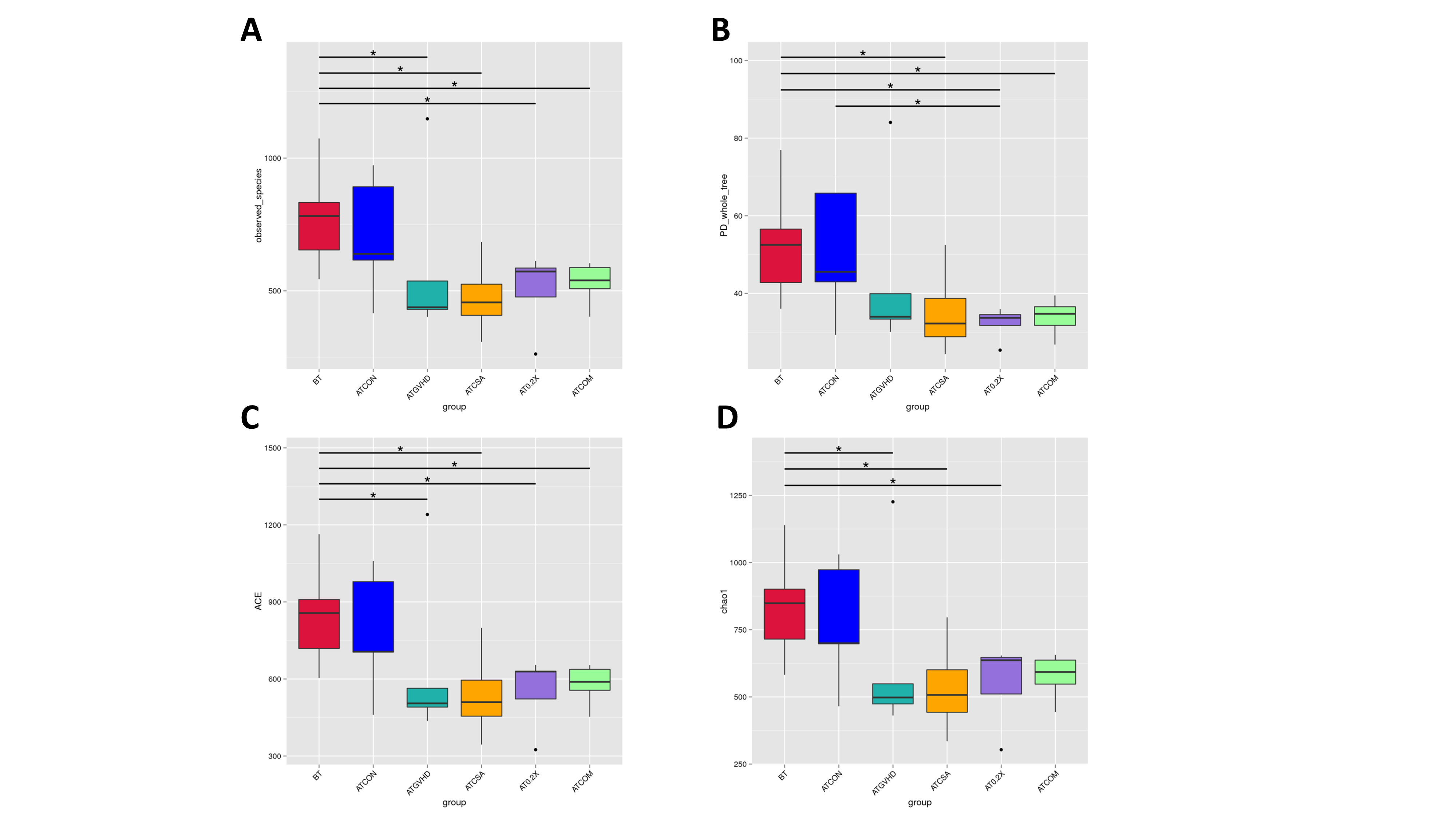

Supplement: Supplementary Figure 2 — Alpha diversity indexes of different groups the 16S rRNA sequencing. (A) Observed species index. OTU numbers in aGVHD mice were significantly decreased. (B) PD whole tree index. Species’ genetic relationship in aGVHD mice becomes simple. (C) ACE index. (D) Chao1 index. Panels (C,D) revealed that species numbers were significantly reduced in aGVHD mice. n = 4–6/group, *p < 0.05, **p < 0.01. [file Image_2.tif]

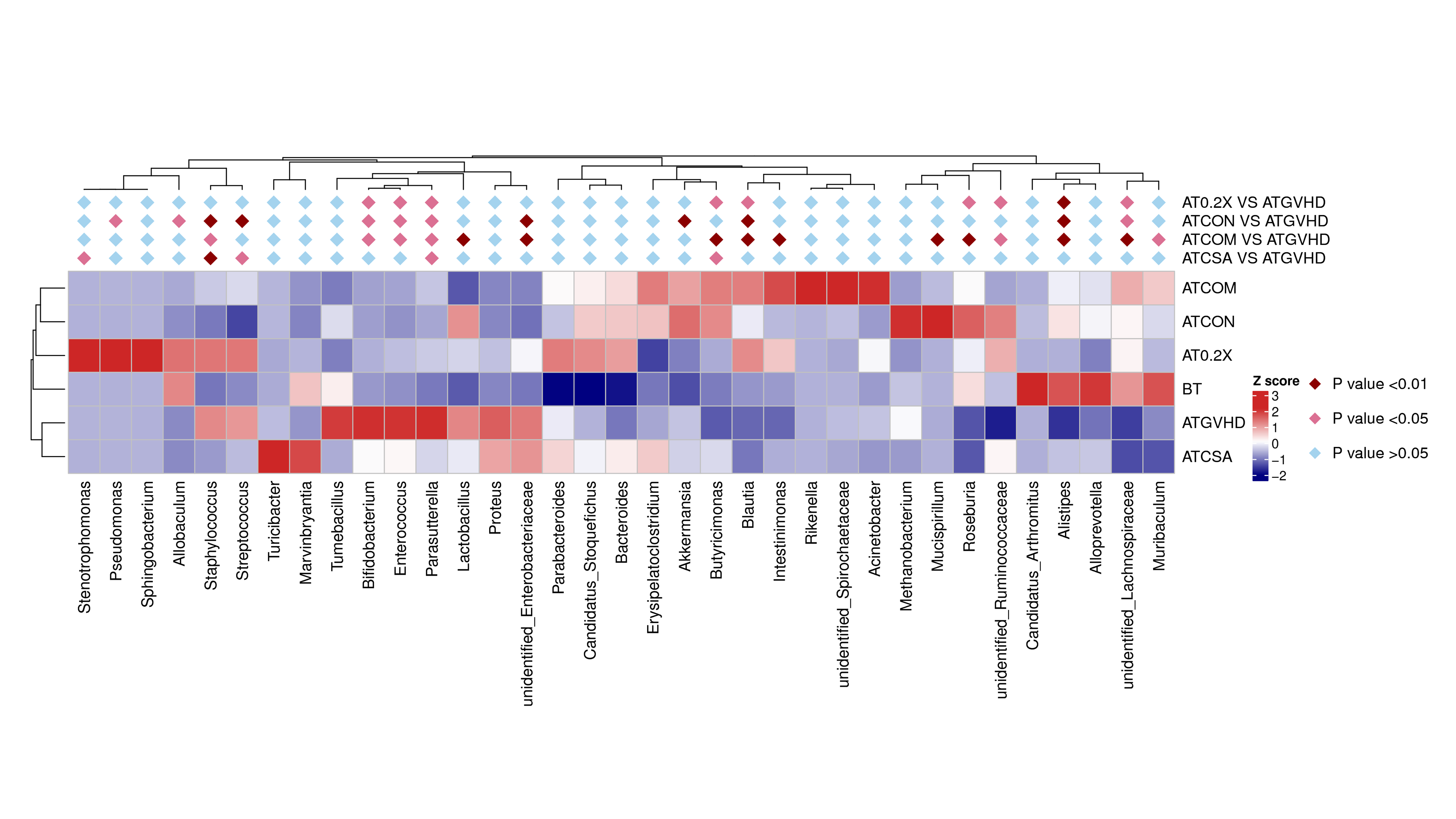

Supplement: Supplementary Figure 3 — The statistic analysis of the abundance of top 35 genera between different groups of mice. The heat map is color-based on row Z scores. The highest and lowest bacterial abundance in each genus was marked with red and blue, respectively. The combination treatment significantly up-regulated the relative abundance of Alistipes, unidentified Ruminococcaceae, and Akkermansia. The relative abundance of Enterobacteriaceae and Enterococcus were down-regulated in mice receiving the combo treatment. n = 4–6/group. [file Image_3.tif]

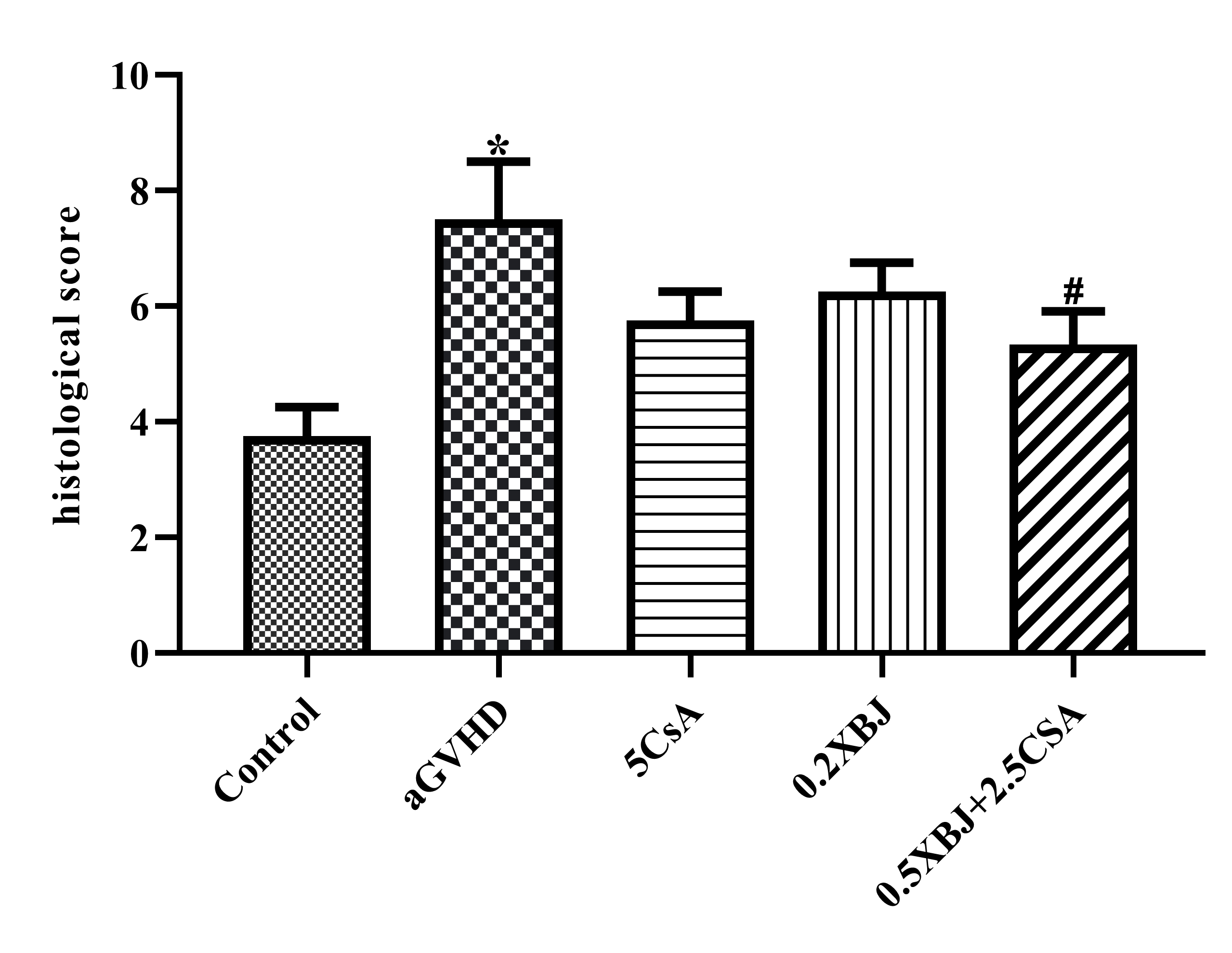

Supplement: Supplementary Figure 4 — Histopathological scores of colons in different groups of mice. n = 4–6/group, *p < 0.05, Control vs aGVHD group; #p < 0.05, compared with aGVHD group. [file Image_4.tif]
